# PESIN Conjugates for Multimodal Imaging: Can Multimerization Compensate Charge Influences on Cell Binding Properties? A Case Study †

**DOI:** 10.3390/ph14060531

**Published:** 2021-06-02

**Authors:** Ralph Hübner, Alexa Paretzki, Valeska von Kiedrowski, Marco Maspero, Xia Cheng, Güllü Davarci, Diana Braun, Helen Damerow, Benedikt Judmann, Vasileios Filippou, Clelia Dallanoce, Ralf Schirrmacher, Björn Wängler, Carmen Wängler

**Affiliations:** 1 Biomedical Chemistry, Department of Clinical Radiology and Nuclear Medicine, Medical Faculty Mannheim of Heidelberg University, Theodor-Kutzer-Ufer 1-3, 68167 Mannheim, Germany; marco.maspero@medma.uni-heidelberg.de (M.M.); Diana.Braun@medma.uni-heidelberg.de (D.B.); Helen.Damerow@medma.uni-heidelberg.de (H.D.); Benedikt.Judmann@medma.uni-heidelberg.de (B.J.); 2 Institute of Inorganic Chemistry, University Stuttgart, Pfaffenwaldring 55, 70550 Stuttgart, Germany; a.paretzki@gmx.net (A.P.); filippou@varimol.de (V.F.); 3 Molecular Imaging and Radiochemistry, Department of Clinical Radiology and Nuclear Medicine, Medical Faculty Mannheim of Heidelberg University, Theodor-Kutzer-Ufer 1-3, 68167 Mannheim, Germany; valeska.vonkiedrowski@medma.uni-heidelberg.de (V.v.K.); Xia.Cheng@medma.uni-heidelberg.de (X.C.); Guellue.Davarci@medma.uni-heidelberg.de (G.D.); Bjoern.Waengler@medma.uni-heidelberg.de (B.W.); 4 Department of Pharmaceutical Sciences, Medicinal Chemistry Section “Pietro Pratesi”, University of Milan, Via L. Mangiagalli 25, 20133 Milan, Italy; clelia.dallanoce@unimi.it; 5 Department of Oncology, Division of Oncological Imaging, University of Alberta, 11560 University Avenue, Edmonton, AB T6G 1Z2, Canada; schirrma@ualberta.ca

**Keywords:** cell binding, copper-64, gallium-68, GRPR affinity, indocyanine dyes, multimodal imaging, NIR fluorescent dyes, PESIN multimers

## Abstract

Recently, anionic charges were found to negatively influence the in vitro gastrin-releasing peptide receptor (GRPR) binding parameters of dually radioisotope and fluorescent dye labeled GRPR-specific peptide dimers. From this, the question arose if this adverse impact on in vitro GRP receptor affinities could be mitigated by a higher valency of peptide multimerization. For this purpose, we designed two different hybrid multimodal imaging units (MIUs), comprising either one or two click chemistry-compatible functional groups and reacted them with PESIN (PEG_3_-BBN_7–14_, PEG = polyethylene glycol) dimers to obtain a dually labeled peptide homodimer or homotetramer. Using this approach, other dually labeled peptide monomers, dimers, and tetramers can also be obtained, and the chelator and fluorescent dye can be adapted to specific requirements. The MIUs, as well as their peptidic conjugates, were evaluated in terms of their photophysical properties, radiolabeling efficiency with ^68^Ga and ^64^Cu, hydrophilicity, and achievable GRP receptor affinities. Here, the hydrophilicity and the GRP receptor binding affinities were found to be especially strongly influenced by the number of negative charges and peptide copies, showing log*_D_* (1-octanol-water-distribution coefficient) and IC_50_ (half maximal inhibitory concentration) values of −2.2 ± 0.1 and 59.1 ± 1.5 nM for the homodimer, and −1.9 ± 0.1 and 99.8 ± 3.2 nM for the homotetramer, respectively. From the obtained data, it can be concluded that the adverse influence of negatively charged building blocks on the in vitro GRP receptor binding properties of dually labeled PESIN multimers can, at least partly, be compensated for by the number of introduced peptide binding motives and the used molecular design.

## 1. Introduction

The non-invasive imaging of diseases, especially for the delineation and staging of cancer, visualization of tissue function, and detection of inflammation and other pathological changes, is important for the sensitive and correct diagnosis and adequate therapy of patients [1,2,3,4,5,6]. Functional imaging, e.g., by highly sensitive imaging techniques such as positron emission tomography (PET) or single photon emission computed tomography (SPECT), plays an essential role, as these modalities can be used to visualize diseases very sensitively and specifically at a very early stage [7,8,9]. Within the last few years, multimodal imaging has increasingly become a focus of clinical research, as it allows the advantages of different modalities to be combined [10,11,12,13,14]. Further advantages are the improved efficiency, higher quality of the obtained results by gathering complementary information, and cross-validation of the imaging results.

One particularly interesting multimodal imaging approach is PET/OI (optical imaging), enabling pre-operative whole-body imaging, e.g., of malignancies by PET, followed by intraoperative fluorescence-guided surgery or in-line biopsy by OI [13,15,16,17,18,19,20]. For this purpose, tumor-specific imaging agents have to be developed which enable the specific and sensitive visualization of the target malignancies by both modalities. Although it is of course possible to use different individual imaging agents for PET and OI, the use of hybrid multimodal imaging agents that can be detected by both modalities has some advantages. A combined agent, accumulating specifically in the target tissue and showing a high retention there, enables performing pre-operative imaging, visualizing all tumor material, and efficiently delineating it during the following surgery. This is especially favorable in case of malignantly transformed lymph nodes, surrounding small metastases, and infiltratively growing tumors, where it can be difficult to determine the tumor margins. Different imaging probes, each being only detectable by PET or OI, can exhibit different pharmacokinetic profiles, as well as different tumor visualization potentials. This leads to a dissimilar biodistribution pattern, adding a degree of uncertainty to the clinical imaging results.

Hence, besides developing and improving multimodal imaging modalities, the development of hybrid multimodal imaging probes has also gained increasing interest over the last few years [10,15,16,21]. Within the group of dually radionuclide and fluorescent dye labeled agents, peptide-based compounds are particularly interesting, as they combine the favorable properties of peptides, such as straightforward chemical synthesis and modification, fast pharmacokinetics, efficient tissue penetration, high target affinity, and specific accumulation in target tissues, with those mentioned before. As peptidic agents are relatively small, molecular changes can however result in a significant alteration of their biological activity, especially when two reporter units for imaging (radionuclide and fluorescent dye) are introduced independently in different positions of the peptide carrier [4,21,22,23,24,25].

Thus, attempts have been made within the few last years to develop combined multimodal imaging units (MIUs) which comprise both, a chelator/radiolabel, and a fluorescent dye and that can be introduced efficiently into peptidic carriers with the aim of altering the receptor binding profile of the modified peptides as little as possible [26,27].

Regarding the influence of MIU introduction into peptides, it was found in previous studies [26,27] that the conjugation of different fluorescent dyes and NODA-GA chelator-comprising hybrid synthons to a PESIN homodimer did not per se alter the properties of the resulting bioconjugates, in terms of receptor affinity (NODA-GA = 1,4,7-triazacyclononane,1-glutaric acid-4,7-acetic acid, PESIN = PEG_3_-BBN_7–14_, BBN_7–14_ = truncated peptide sequence of the endogenous GRPR ligand bombesin). In these systems, neither the photophysical properties of the used dyes nor the radiolabeling of the MIUs or the conjugates were affected by the complex molecular designs. In contrast, the compound hydrophilicity and GRPR affinity were, in part, considerably influenced by the used fluorescent dye, and especially its charge, requiring the tailoring of the properties of the agents. In detail, it was found that the higher the number of negative charges of the used fluorescent dye, the lower was the lipophilicity of the homodimers and also the GRPR binding affinity. This is in accordance with earlier studies on a fluorescent dye-modified BBN_7–14_ monomer, showing a five-fold reduction in GRPR affinity when replacing a neutrally charged carbocyanine dye by a two-fold negatively charged fluorescein [28].

**Figure 1:**
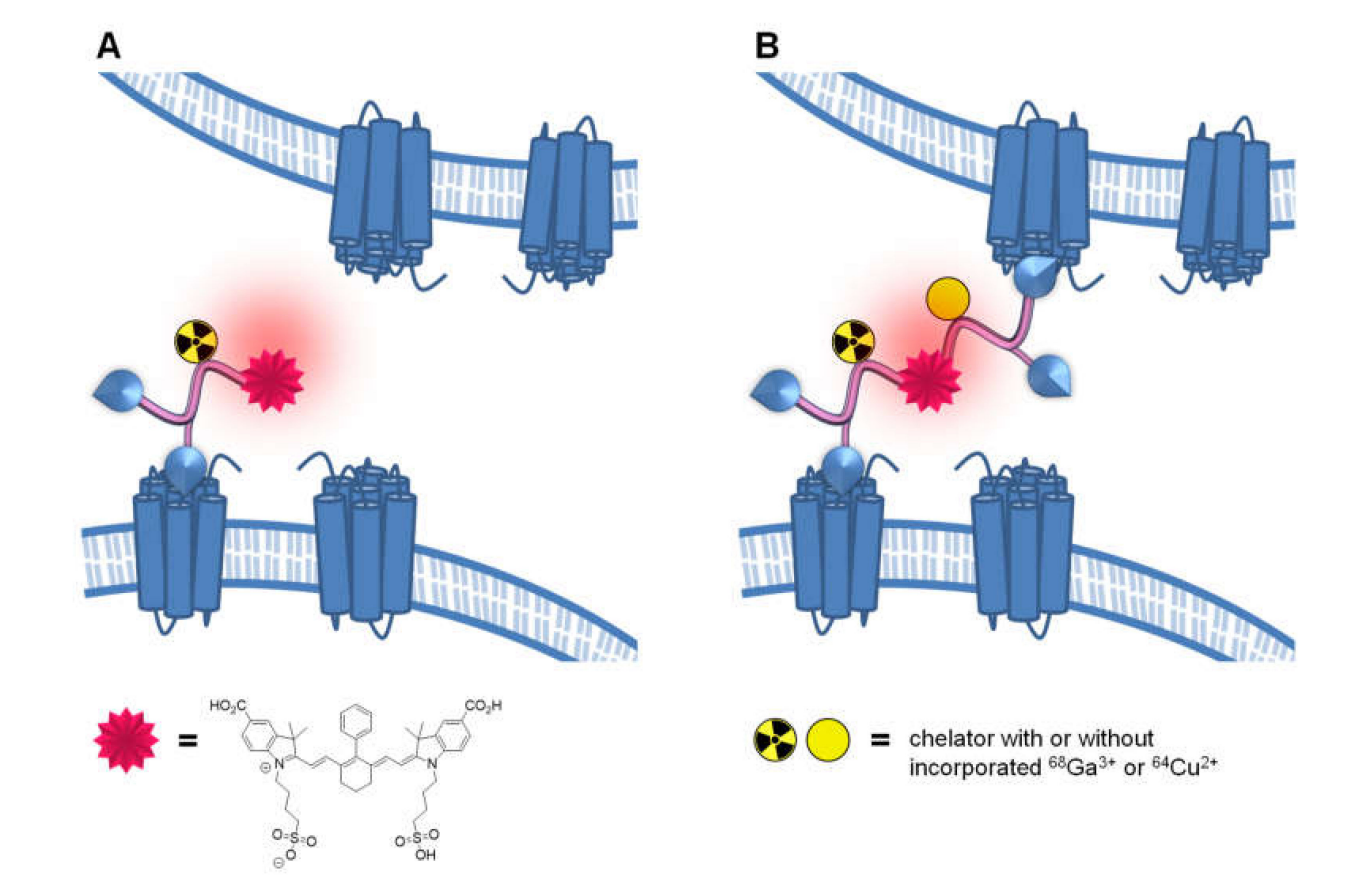
Schematic presentation of the study concept, investigating the influence of a negatively charged hybrid multimodal imaging unit in a PESIN dimer and tetramer on the GRPR binding parameters of the respective bioconjugates. Peptide multimers can result in higher target affinities than monomers, as they can bind with higher probability to the receptor, due to the forced proximity effect (**A**), or by simultaneous binding (**B**). The developed synthetic approach for the peptide multimers builds on a MIU design utilizing the NIR fluorescent dye as a symmetrical branching unit for the assembly of multimers.

Thus, at least for GRPR-binding peptides, the effect of negative charges in direct vicinity to the receptor-affine peptide motive was shown to be relevant for the receptor interaction of peptide monomers and also dimers. However, the question remains if this is also the case when studying multimers of a higher valency. In principle, two different scenarios are conceivable: (i) that the GRPR binding affinity of the homomultimer is diminished by the introduction of a negatively charged dye and chelator near the receptor-binding sequence, as was found for the homodimer; or (ii) that the affinity is not influenced to such a considerable extent when a higher number of receptor-affine peptide copies is present in the molecule, meaning that the higher number of peptides per molecule can compensate for the effect exerted by the negative charges of the dye and chelator. In order to determine which of these scenarios applies, we directly compared a MIU-modified homodimer and homotetramer of the GRPR-binder PESIN, comprising the same, negatively charged near-infrared (NIR) fluorescent dye (Figure 1).

## 2. Results and Discussion

### 2.1. Synthesis of the Hybrid Multimodal Imaging Units **2** and **3** and Their Peptide Multimer Conjugates **5** and **6**

**Scheme 1:**
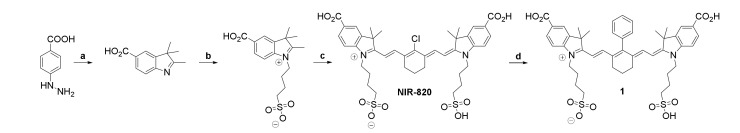
Schematic depiction of the synthetic pathway towards the used NIR-820 derivative **1**. Reagents and conditions: (**a**) 3-methyl-2-butanone, acetic acid, sodium acetate, 5 h, reflux, 90% yield; (**b**) 1,4-butansultone, 1,2-dichlorobenzen, 8 h, 160 °C, 90% yield; (**c**) N-((2-chloro-3-((phenylimino)methyl)cyclohex-2-en-1-ylidene)methyl)aniline, acetic acid, acetic anhydride, sodium acetate, 45 min, reflux, strictly inert conditions, 45% yield; (**d**) phenylboronic acid; Pd(PPh_3_)_4_, 80% yield.

As a conceptual basis for the MIUs studied here, as well as their peptide conjugates, the fluorescent dye NIR-820 (Scheme 1) was used, as it comprises negative charges whose influence on the conjugated peptides moieties was to be studied. The two carboxylate moieties contained enabled the design of peptide dimers, as well as tetramers, by further modifying only one, or both, carboxylic functionalities with the chelator NODA-GA and PESIN peptide dimers. Thus, the presented modular synthesis pathway is also adaptable to the assembly of other peptide monomers and multimers.

NIR-820 was synthesized according to published protocols [29,30,31] and further modified in the central position with a phenyl group, by a Suzuki cross-coupling reaction using [Pd(PPh_3_)_4_] in water to generate a robust C-C bond in this position of the molecule [30,31,32,33] (Scheme 1). This was necessary as the central chlorine atom, as well as the respective *N*- or *S*-substituted derivatives, are very sensitive to nucleophilic attack and further substitution [32,34,35,36,37].

**Scheme 2:**
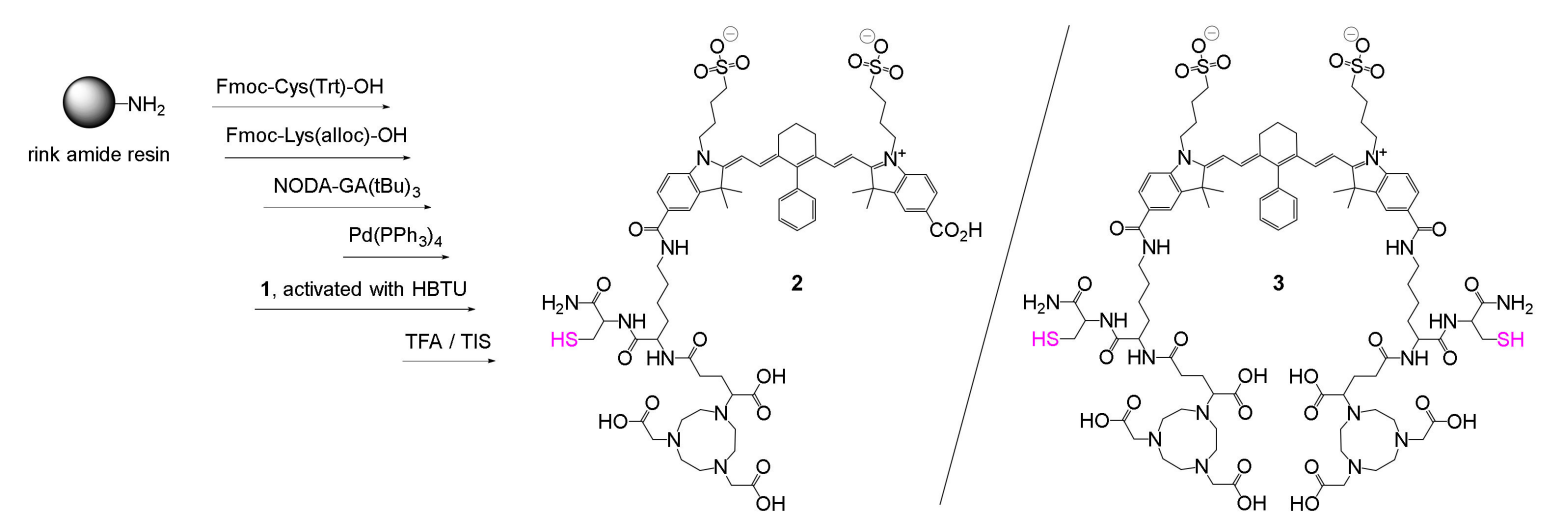
Schematic depiction of the synthesis pathway towards the thiol-, NODA-GA chelator-, and NIR dye-comprising MIUs **2** and **3,** which were obtained by solid phase-based synthesis. Thiol groups that can be reacted with arbitrary maleimide-modified biomolecules are depicted in magenta. Reagents and conditions: for conjugation of Fmoc-Cys(Trt)-OH and NODA-GA(*t*Bu)_3_: resin swelling 45 min, Fmoc-deprotection: piperine/DMF (1:1), 5 min, amino acid conjugation: 4 equiv. activated amino acid for 30–60 min (activation of Fmoc-Cys(Trt)-OH: 3.9 equiv. HBTU, 4 equiv. DIPEA, 2 min, activation of NODA-GA(*t*Bu)_3_: 1.95 equiv. HBTU, 2 equiv. DIPEA, 2 min); alloc deprotection: 1.5 equiv. Pd(PPh_3_)_4_ and 10 equiv. morpholine in CH_2_Cl_2_ for 2 h under absence of light; dye conjugation: see Materials and Methods section for optimal dye to resin ratios, 80 °C for 3–4 h; cleavage from resin and deprotection: TFA/TIS (95:5 *v*/*v*); 90 min; 20% yield for **2**, 20% yield for **3**. DMF = *N*,*N*-dimethylformamide, HBTU = (2-(1*H*-benzotriazol-1-yl)-1,1,3,3-tetramethyluronium hexafluorophosphate, DIPEA = *N*,*N*-diisopropylethylamine, TIS = triisopropylsilane, TFA = trifluoroacetic acid.

From dye **1**, the hybrid MIUs **2** and **3** were synthesized according to standard solid phase peptide synthesis protocols, starting from a rink amide resin, which was first reacted with cysteine to introduce the maleimide-reactive cysteine used for bioconjugation, and second with lysine, to which in the *N*_α_-position, the chelator NODA-GA was attached. This lysine was further reacted in the *N*_ε_-position with **1** to give MIU **2** (Scheme 2).

Using this approach, not only the hybrid agent **2** was obtained, using standard amounts of four equivalents of one-fold activated dye per amino functionality, but always a mixture of **2** and **3**. Although a small amount of **3** was expected to be formed due to the partial double-activation of **1**, the ratio between both products was always about 1:1. In order to identify the optimal reaction parameters to obtain **2** as the major product, several reaction conditions were tested; among others, different loadings of the rink amide resin (0.19, 0.54, and 0.71 mmol/g), as well as different concentrations of the dye molecule (0.5, 1, 2, 4, and 8 equiv. of dye per amino functionality on resin), different activation reagents (HBTU, HATU (*N*,*N*,*N*′,*N*′-tetramethyl-*O*-(7-azabenzotriazol-1-yl)uronium-hexafluorphosphate) and PyBOP (benzotriazole-1-yl-oxy-tris-pyrrolidino-phosphonium hexafluorophosphate)), and highly diluted dye solutions were tested. None of these attempts however resulted in a shift in the ratio between both products in favor of **2**, and the ratio between products **2** and **3** remained at about 1:1. Further tests, applying milder conditions, such as reaction at ambient temperature for dye conjugation, did not result in product formation at all, and reducing the time for conjugation decreased the amount of formed product for both agents **2** and **3**.

**Figure 2:**
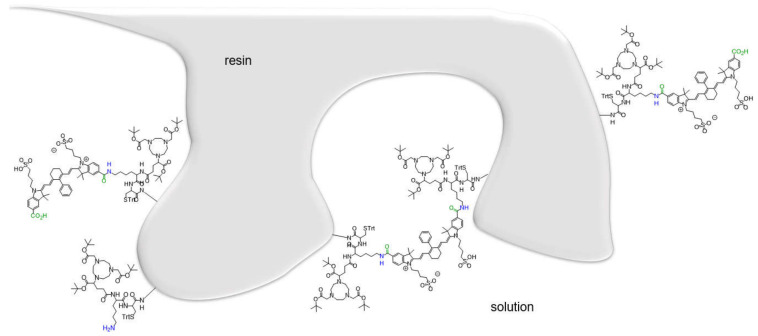
Schematic depiction of the assumed reason for the observed double reaction of **1**, giving both MIUs **2** and **3** in the same reaction step. In positions with only one isolated functional reactive amino group on the resin (blue), **2** is formed as the product (left and right), whereas in positions with two amino groups in the direct vicinity, a twice-activated dye can react on both ends (reactive carboxyl groups are depicted in green), resulting in 3 after cleavage and deprotection (middle).

It can be assumed that the formation of **3** is likely to be a result of the folded surface of the resin (Figure 2), enabling the twofold reaction of the dye, although stoichiometrically, and only one of the carboxylic acids should be present in the activated form (meaning, however, that a mixture of non-activated, once, and twice activated dyes is present in the mixture).

**Scheme 3:**
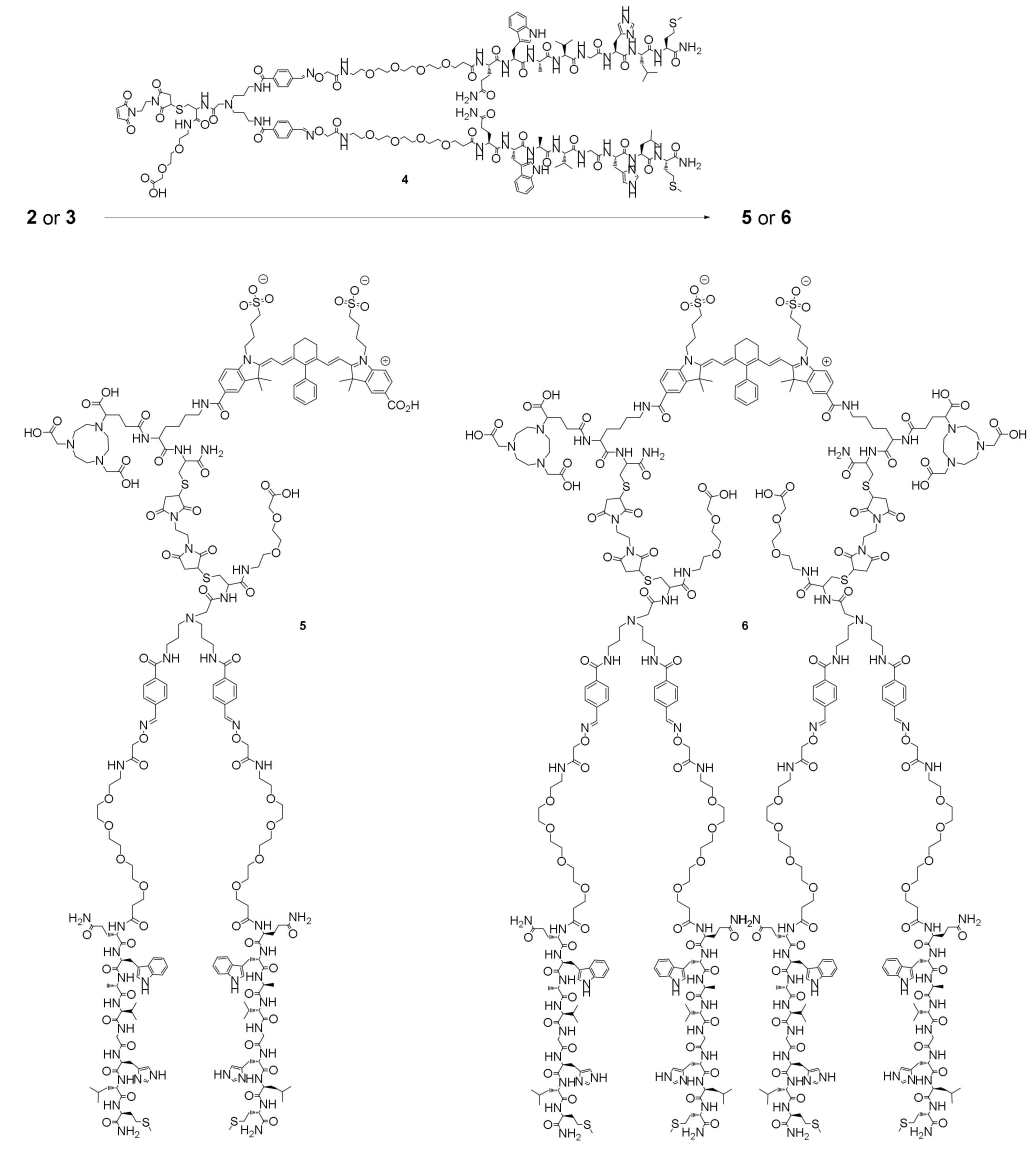
Schematic depiction of the synthesis pathway to the target hybrid, chelator, and fluorescent dye-modified PESIN homodimer **5** and homotetramer **6**. Reagents and conditions: H_2_O:MeCN 1:1 (*v*/*v*) + 0.1% TFA + phosphate buffer (0.5 M, pH 7.2), pH 7.0, 5 min, ambient temperature, 40% yield for **5**, 35% yield for **6**.

After the separation and purification of both MIUs **2** and **3**, both agents were further reacted with a maleimide-modified PESIN dimer **4** (Scheme 3), which was synthesized as previously described [26,27]. The reaction was carried out under mild conditions via the bioorthogonal Michael addition click reaction [38] and produced the target products **5** and **6** after 5 min reaction time (Scheme 3).

This approach of using the described modular system to synthesize the multimodal target agents allows the compounds to be adapted to specific requirements and thus enables a tailored molecular design in which individual parts of the molecule, such as the chelator, dye, and peptide, can be easily exchanged.

### 2.2. Determination of Photophysical Properties of Dyes NIR-820 and **1**, MIUs **2** and **3,** and Their Peptide Multimer Conjugates **5** and **6**

After successful synthesis of the target compounds **5** and **6**, we studied the photophysical properties of all agents. We intended to rule out a negative influence of the conversion of the dye (**1**) to the MIUs (and here, especially to the two-fold reacted species **3**) or of the conjugation to the complex peptidic structures (in **5** and **6**) on the absorption coefficients, or the absorption or emission wavelengths. The results of the studies are summarized in Table 1 and show a reduction of the absorption coefficient upon conjugation of **1** that is within the usually observed range for such agents, whereas no considerable shift of the absorption or emission maxima was observed. The conjugates did not show different results between the one- or two-fold derivatized agents, and no further changes of the absorption or emission characteristics were observed upon conjugation to the peptidic units.

### 2.3. Radiolabeling of **2**, **3**, **5**, and **6** and Determination of Lipophilicity/Hydrophilicity of [^68^Ga]Ga-**2**, [^68^Ga]Ga-**3**, [^68^Ga]Ga-**5**, and [^68^Ga]Ga-**6**

In the following, the MIUs **2** and **3**, as well as their bioconjugates **5** and **6**, were radiolabeled with the positron emitters ^68^Ga^3+^ (t_1/2_ = 68 min) and ^64^Cu^2+^ (t_1/2_ = 12.7 h) in order to show their applicability in combined hybrid multimodal imaging using PET and OI. Labeling with ^68^Ga was performed at 45 °C for 10 min using standard acetate buffered conditions at pH 4.0 [40]. For ^64^Cu, the labeling was performed at ambient temperature for 10 min using the same buffer system but with another pH of 5.0, to achieve efficient radiometal incorporation [41]. In order to obtain pure radiolabeled products, ascorbic acid had to be added to the labeling mixtures in order to avoid the disintegration of the large carbocyanine dye systems that are susceptible to the radiolysis-induced formation of ions and radicals [42]. The labeling reactions of the MIUs **2** and **3** required the addition of *tris*(2-carboxyethyl)phosphine (TCEP) as a reducing agent to the labeling mixtures. If omitted, the formation of the labeled MIUs, as well as their dithiol-bridged dimers, was observed [26,27]. Under these conditions, the labeled products [^68^Ga]Ga-**2**, [^68^Ga]Ga-**3**, [^68^Ga]Ga-**5**, and [^68^Ga]Ga-**6** could be obtained in high radiochemical yields and purities of >95%. The respective ^64^Cu-labeling also resulted in complete radiometal incorporation into the precursor molecules but gave the products lower radiochemical purities of >90%, as no ascorbic acid could be used as a radical scavenger. The ascorbic acid was found to act as a complex ligand itself, resulting in decreased ^64^Cu-NODA-GA incorporation rates or molar activities when more precursor was used. Being unable to use ascorbic acid as scavenger resulted in the slow decomposition of the susceptible dye molecules in the radiolabeling solution and lower radiochemical purities of >90%. The products were obtained in non-optimized molar activities of 5–40 GBq/µmol for [^64^Cu]Cu-**2**, [^64^Cu]Cu-**3**, [^64^Cu]Cu-**5**, and [^64^Cu]Cu-**6**, and 18–60 GBq/µmol for [^68^Ga]Ga-**2**, [^68^Ga]Ga-**3**, [^68^Ga]Ga-**5**, and [^68^Ga]Ga-**6**.

For the ^68^Ga-labeled agents [^68^Ga]Ga-**2**, [^68^Ga]Ga-**3**, [^68^Ga]Ga-**5**, and [^68^Ga]Ga-**6**, and the dyes NIR-820 and **1**, the log*_D_* values were determined by partition experiments between *n*-octanol and aqueous solution at pH 7.4. As expected from preceding studies [26,27], the MIUs [^68^Ga]Ga-**2** and [^68^Ga]Ga-**3** exhibited relatively high hydrophilicities of −2.6 ± 0.1 and −2.9 ± 0.1, respectively, similar to those of the unconjugated dyes NIR-820 (−3.3 ± 0.7) and **1** (−3.2 ± 0.5). By conjugation of the MIUs to **4**, the resulting homodimer [^68^Ga]Ga-**5** and homotetramer [^68^Ga]Ga-**6** showed the expected decrease in hydrophilicity, being caused by the conjugation of the peptides. As the PESIN homodimer alone exhibits a considerably lower hydrophilicity than the MIUs, with a log*_D_* value of −1.0 ± 0.0 [27], it is furthermore not surprising that [^68^Ga]Ga-**6** (log*_D_* of −1.8 ± 0.1) demonstrated an even lower hydrophilicity compared to [^68^Ga]Ga-**5** (log*_D_* of −2.2 ± 0.1).

### 2.4. Determination of In Vitro GRPR Binding Affinities of **5** and **6**

Since the main focus of the study was on whether it is possible to reduce the adverse influence of negatively charged dyes on the in vitro GRP receptor binding behavior of the corresponding peptidic conjugates by assembling the hybrid multimodal agents on the basis of a higher peptide valency, the bioconjugates [^68^Ga]Ga-**5** and [^68^Ga]Ga-**6** were investigated with respect to their GRPR affinity. For this purpose, we conducted competitive displacement assays on stably GRPR-transfected HEK-293 cells, using [125]I-Tyr4-bombesin as a competitor and endogenous bombesin (BBN) as an internal standard with a known high affinity for this receptor type.

**Figure 3:**
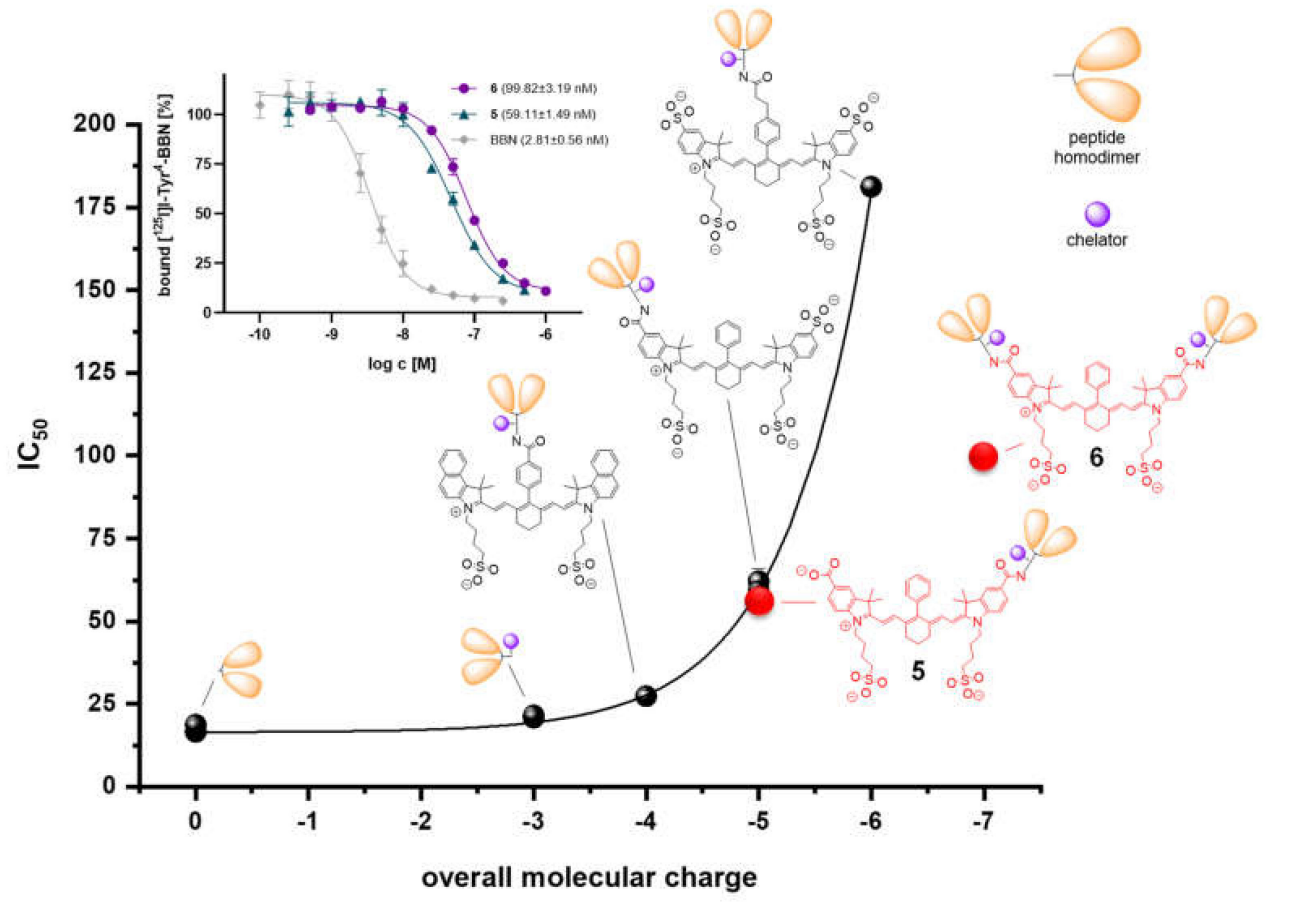
Depiction of the results of the competitive displacements assays on GRPR-transfected HEK-293 cells for in vitro determination of GRPR affinities of the new hybrid multimodal imaging agents **5** and **6**. Graphical classification of the new data (red data points and structures) in the context of the literature values (black data points and structures). Shown is the correlation between IC_50_ values and overall molecular charge of the MIU-derivatized PESIN dimers (black data points and **5**) and tetramer (**6**). Insert: binding curves, giving IC_50_ values of 2.8 ± 0.6 nM for bombesin, 59.1 ± 1.5 nM for **5** and 99.8 ± 3.2 nM for **6**. Results were obtained from at least three different experiments, each performed in triplicate.

For **5**, an IC_50_ value of 59.1 ± 1.5 nM was determined, in line with the results obtained for previously studied agents featuring the same molecular composition, of a PESIN dimer functionalized with MIUs comprising a NODA-GA chelator and an NIR fluorescent dye. In those studies, similar carbocyanine dyes were used, bearing different numbers of sulfonate groups, and thus different numbers of negative charges, of −1, −2, or −3, respectively (Figure 3) [27]. A direct correlation between the number of negative charges and the loss of GRPR binding affinity was found, which prompted us to conduct the study presented here. The IC_50_ value obtained for dimer **5** (exhibiting a charge of −2 for the used dye and an overall molecular charge of −5) perfectly aligned with our expectations (Figure 3), confirming the major role of negative charges in GRPR affinities.

This observed adverse influence of negatively charged dyes on the GRPR affinities of the peptide conjugates might have been a result of coulomb repulsion between the negatively charged MIUs and the receptor protein, which also exhibited several negative charges within its extracellular loops [43]. This rather speculative explanation could in the future be proven by appropriate molecular modeling and docking studies.

Another explanation could be that negatively charged dyes per se (by an unknown mechanism) adversely affect peptide-receptor-interactions, similarly to in previous reports [26,27]. Comparable results were found for a negatively charged FITC-modified BBN_7–14_ peptide and the corresponding neutrally charged NIR dye analog (vide supra), as well as for the respectively modified Tyr^3^-octreotate analogs [28], binding to somatostatin receptors (SSTR) instead of GRPR. In this study, it was not only found that the negatively charged FITC dye resulted in an approximately two-fold lower affinity of the peptide conjugate to SSTR-positive cells than the neutral carbocyanine dye, but that a positively charged analog of the NIR dye even resulted in a roughly five-fold higher affinity than using the neutral derivative. This effect, that positively charged dyes can have an even slightly positive effect on in vitro receptor interactions of the respective bioconjugates, was also confirmed by our own preceding work, concerning other conjugates and studying a positively charged pyrylium dye-modified PESIN homodimer [26].

However, not only the in vitro receptor interaction of dye-peptide-bioconjugates seems to be adversely influenced by using negatively charged dyes, but also the in vivo pharmacokinetic profile of the respective agents. Achilefu et al. showed low tumor uptakes and suboptimal tumor-to-background ratios for their negatively charged FITC-Tyr^3^-octreotate bioconjugate discussed above but significantly better results for the corresponding neutrally charged cyanine dye-modified analog, which enabled excellent tumor visualization by OI in a SSTR-positive tumor mouse model [28], although the latter showed only a two-fold higher SSTR affinity.

On the basis of the results discussed previously, a relatively high IC_50_ value of about 400–500 nM was expectable for PESIN tetramer **6**, as this agent comprises an overall molecular charge of −7; six of these resulting from the carboxylates of the NODA-GA chelators and one resulting from the dye molecule. In addition, as it was shown before that PESIN tetramers show comparably lower GRPR affinities than dimers, due to missing receptor clustering, increased molecular size, complexity, and entropy, reducing the probability of receptor interaction of the tetramer compared to the dimer [44], a further reduction of affinity of **6** compared to **5** was considered to be possible.

In contrast, **6** showed an experimental IC_50_ value of 99.8 ± 3.2 nM, being lower than the affinity found for **5**, but considerably higher than expected (Figure 3).

An explanation for this could be that the number of negative charges being brought into the molecule by the NODA-GA chelator(s) and the dye does not have as strong an effect on the GRPR affinity in case of **6** as it does for **5**, due to the twice as high number of present peptide copies in **6** compared to **5**. The four peptide copies could lead to a spatial shielding of the negative charges (especially as the NODA-GA chelators and the dye are located in the center of **6,** whereas they are more peripherally located in **5**), and the histidine residues of the peptides could be partially protonated under physiological conditions in this complex molecule [45,46], in part compensating for the negative charges. This second effect would also be more pronounced in case of a higher number of peptide moieties per molecule, and thus in **6**.

Taken together, we were able to conclusively corroborate that anionic charges of a fluorescent dye and chelator being conjugated to a peptide dimer specifically targeting GRP receptors considerably affect the receptor affinities of the resulting conjugates.

Additionally, we were able to demonstrate that, besides the net charge of the MIU motive, the structural composition and the number of peptidic binding motives present in the molecule are also of relevance for the observed receptor affinities, apart from multivalent receptor binding.

Thus, the adverse influence of negatively charged building blocks on GRP receptor binding affinities of dually labeled peptide multimers can, at least partly, be compensated for by the number of included peptide binding motives.

## 3. Materials and Methods

Genera*l.* If not otherwise stated, all commercially available solvents and chemicals were used without further purification. Resin, Fmoc-protected amino acids, HATU, PyBOP and polyethylenglycol linkers (Fmoc-NH-PEG_1_-OH and Fmoc-NH-PEG_3_-OH) were purchased from Novabiochem (Darmstadt, Germany), Carbolution (St. Ingbert, Germany) and Iris Biotech (Marktredwitz, Germany), respectively, *N*-succinimidyl-4-formylbenzoate (SFB) from abcr (Karlsruhe, Germany), piperidine, HBTU and TFA, as well as water for HPLC and DMF, from Carl Roth (Karlsruhe, Germany). DIPEA, *N*-[(3-(anilinomethylen)-2-chloro-1-cyclohexen-1-yl)methylen]anilin-monohydrochlorid and TIS were obtained from Sigma-Aldrich (Darmstadt, Germany), 4-hydrazinobenzoic acid, phenylboronic acid, 1,2-*bis*(maleimido)ethane (BME) and *tetrakis*(triphenylphosphine)palladium(0) (Pd(PPh_3_)_4_) from TCI (Eschborn, Germany), and 4-(4,7-*bis*(2-(*tert*-butoxy)-2-oxoethyl)-1,4,7-triazacyclononan-1-yl)-5-(tert-butoxy)-5-oxopentan-oic acid ((*R*)-NODA-GA(tBu)_3_) from CheMatech (Dijon, France).

NIR-820 [47] and its modified derivative **1** [31,39], PESIN [36] and its maleimide-modified homodimer **4** [26] were synthesized according to literature methods. For solid phase-based syntheses, Fmoc-based solid phase peptide synthesis protocols were applied [44]. In brief, the couplings were carried out in DMF for 30 min using 4 equiv. of respective amino acid and 3.9 equiv. of HBTU as the coupling reagent with 4 equiv. DIPEA as the base. Removal of the Fmoc protecting groups was achieved using 50% piperidine in DMF (*v*/*v*) (2 + 5 min), the removal of the allyloxycarbonyl protecting group was achieved by using Pd(PPh_3_)_4_ in dichloromethane (DCM) and morpholine as base for 2 h. Cleaving from solid support and deprotection of acid-labile protecting groups was accomplished using TFA:TIS (95:5 (*v*/*v*)) for 60–90 min, the volatile components of the product containing cleavage mixtures were evaporated to dryness under reduced pressure, and the products were purified by semipreparative HPLC. After lyophilization, the compounds were obtained as white or green (dye containing) solids.

For analytical and semipreparative HPLC, Dionex Ultimate 3000 systems (Chromeleon Software, version 6.80) (Dreieich, Germany) were used together with analytical Chromolith Performance (RP-18e, 100–4.6 mm) or semipreparative Chromolith Semiprep (RP-18e, 100–10 mm) columns and H_2_O and MeCN + 0.1% TFA as solvents at a flow rate of 4 mL/min. For analysis of radioactive material, an analogous system, additionally equipped with a Raytest GABI Star radioactivity detector was used.

For mass spectrometry and NMR, Finnigan MAT95Q (ESI) (San Jose, CA, USA), Bruker Daltronics Microflex (MALDI) (Bremen, Germany) and Jeol AS500 (NMR) (Peabody, MA, USA) spectrometers were used, respectively. Gamma counting was performed by using a 2480 Wizard gamma counter system from PerkinElmer (Rodgau, Germany). For absorbance and emission experiments, a Tecan Infinite M200 Microplate reader (Männedorf, Switzerland), together with a Nunc Micro-Well 96 plate (ThermoFisher, Dreieich, Germany), was used, whereby the absorbance spectra were additionally recorded with a Cary 100 Bio system from Varian (Darmstadt, Germany) using 4 mL PMMA Cuvettes from Sigma-Aldrich (Darmstadt, Germany).

Human embryonic kidney 293 cells, stably expressing the GRP receptor (GRPR HEK-293 cells), were obtained from Dr. Martin Béhé, Paul Scherrer Institute, Villingen, Switzerland. Cells were cultured using Dulbecco’s modified Eagle’s medium (DMEM, high glucose, GlutaMax-I, 500 mL), geneticin (G418 Sulfate, 50 mg/mL), Opti-MEM I (GlutaMAX I), RPMI 1640 medium and PenStrep from Gibco, fetal calf serum (FCS) from Bio&sell (Feucht, Germany) and Dulbecco’s phosphate buffered saline (PBS), 0.25% Trypsin and 0.02% EDTA solution in PBS from Sigma-Aldrich (Darmstadt, Germany).

[^125^I]I-Tyr^4^ -bombesin was purchased from PerkinElmer (NEX258010UC, molar activity: 81.4 GBq/µmol) (Rodgau, Germany). The ^68^Ge/^68^Ga-Generator used was an IGG100, obtained from Eckert & Ziegler (Berlin, Germany), and eluted by fractioned elution with HCl (0.1 M, 1.6 mL). [^64^Cu]CuCl_2_ solution was obtained from the Department of Preclinical Imaging and Radiopharmacy at the University Hospital Tübingen, Germany.

Synthesis of the hybrid multimodal imaging units **2** and **3**. Rink amide resin-Cys(Trt)-Lys(NODA-GA(tBu)_3_)-NH_2_ was synthesized as previously described [26,36] using standard Fmoc-based solid phase peptide synthesis protocols. **1** was dissolved in DMF (supported by an ultrasonic bath), activated for 2 min with HBTU and DIPEA, and reacted at elevated temperature of 80 °C for 3–4 h with the resin. Afterwards, the resin was filtered and washed with DMF, water, DCM, and diethylether (each step thrice) and dried under reduced pressure. Incubation of the dried resin with TFA:TIS 95:5 (*v/v*) for 90 min was used to cleave the compound from the resin and to remove protecting groups. The volatile components of the resulting mixture were evaporated under reduced pressure and the residue was resolved in H_2_O:MeCN 1:1 (*v*/*v*) + 0.1% TFA and purified by semipreparative HPLC. Both compounds could be isolated in the same reaction, the yields for both molecules were found to be almost independent from the starting ratio between the dye and resin. Analytical data:

**2**: HPLC gradient 25–65% MeCN + 0.1% TFA in 8 min R_t_ = 5.1 min, optimum yield (ratio dye to resin 4:1) 20%, 95% purity. MALDI-MS (*m/z*) for [M+H]^+^ (calculated): 1443.62 (1443.75), for [M + Na]^+^ (calculated): 1466.28 (1466.57), for [M + K]^+^ (calculated): 1482.28 (1482.53); HR-ESI-MS (*m/z*) for [M+Na+K]^2+^ (calculated): 752.7378 (752.7670); **3**: HPLC gradient 25–65% MeCN + 0.1% TFA in 8 min R_t_ = 4.5 min, optimum yield (ratio dye to resin 1:2) 20%, 95% purity. MALDI-MS (*m/z*) for [M+H]^+^ (calculated): 2032.45 (2032.87), for [M + Na]^+^ (calculated): 2054.15 (2054.85), for [M + K]^+^ (calculated): 2070.29 (2070.82); HR-ESI-MS (*m/z*): detection of this compound by HR-ESI-MS was not possible, however, the respective bioconjugate could be successfully identified by HR-ESI-MS.

*General synthesis of the bioconjugates***5***and***6**. For the Michael addition click reaction [38,48] towards **5** and **6**, a mixture of **2** or **3** and **4** (equimolar amounts, 12 µmol) was dissolved in H_2_O:MeCN 1:1 (*v*/*v*) + 0.1% TFA (500 µL) and the pH was adjusted to 7.0 using phosphate buffer (0.5 M, pH = 7.2). After 5 min, the reactions were found to be complete by analytical HPLC, and the products were purified by semipreparative HPLC, giving green solids after lyophilization. Analytical data:

**5**: HPLC gradient 0–100% MeCN + 0.1% TFA in 8 min R_t_ = 5.5 min, 40% yield, 95% purity. MALDI-MS (*m/z*) for [M-H]^−^ (calculated): 4847.41 (4847.16), for [M−2H]^2−^ (calculated): 2422.56 (2422.58); HR-ESI-MS (*m/z*) for [M+Na+K+H]^3+^ (calculated): 1637.3522 (1637.5812); **6**: HPLC gradient 0–100% MeCN + 0.1% TFA in 12 min R_t_ = 6.8 min, 35% yield, 95% purity. MALDI-MS (*m/z*) for [M+K]^+^ (calculated): 8882.17 (8882.35), [M+2H]^2+^ (calculated): 4422.53 (4422.64); HR-ESI-MS (*m/z*): [M+8Na]^8+^ (calculated): 1504.4851 (1504.4932).

*Radiochemistry.* Molar activities of radiotracers were obtained from the activity of the final products per amount of labeling precursor used for radiolabeling. ^64^Cu-labeling: A solution of the respective MIU **2** or **3** or their bioconjugates **5** or **6** (2–5 nmol) in Tracepure^®^ water (2–5 µL) was added to 25–80 MBq of [^64^Cu]CuCl_2_ solution which had been adjusted before to pH 5 by addition of sodium acetate solution (0.25 M, 100 µL). After reaction for 10 min at ambient temperature, the solutions were analyzed by analytical radio-HPLC. The radiolabeling reaction was found to be complete and the products [^64^Cu]Cu-**2**, [^64^Cu]Cu-**3**, [^64^Cu]Cu-**5** and [^64^Cu]Cu-**6** were obtained in radiochemical purities of >90% and non-optimized molar activities of 5–40 GBq/µmol.

^68^Ga-labeling: A solution of the respective MIU **2** or **3** or their bioconjugates **5** or **6** (5 nmol) in Tracepure^®^ water (5 µL) was added to 90–300 MBq of [^68^Ga]GaCl_3_ solution, containing ascorbic acid (2 mg) and adjusted to pH 4.0 by addition of sodium acetate solution (1.25 M, 125 µL). After reaction for 10 min at 45 °C, the solutions were analyzed by analytical radio-HPLC. The radiolabeling reaction was found to be complete and the products [^68^Ga]Ga-**2**, [^68^Ga]Ga-**3**, [^68^Ga]Ga-**5**, and [^68^Ga]Ga-**6** were obtained in radiochemical yields and purities of >95% and non-optimized molar activities of 18–60 GBq/µmol.

*Determination of log_D_.* The *n*-octanol/water partition coefficient (log*_D_*) of [^68^Ga]Ga-**2**, [^68^Ga]Ga-**3**, [^68^Ga]Ga-**5**, and [^68^Ga]Ga-**6** was determined as previously described [27]. In brief, 5 µL of the respective radiolabeled product solutions were added to a mixture of n-octanol (800 µL) and phosphate buffered solution (0.05 M, pH 7.4, 795 µL) and vigorously shaken for 5 min. After separation of the phases by centrifugation, 100 µL of each phase was taken and measured in a γ-counter. For NIR-820 and **1**, the log*_D_* was obtained by comparable *n*-octanol/water partition experiments, followed by HPLC analysis of the phases instead of γ-counting.

Competitive Receptor Binding Assay. The assays were carried out on stably GRPR-transfected HEK-293 cells as described previously [26,27]. In brief, 50 µL of a cell suspension containing 10^5^ cells was seeded in each well of a Millipore 96-well filter plate. To this, a total volume of 50 µL solution was added to each well, containing the GRPR-specific radioligand [^125^I]I-Tyr^4^-bombesin (25 µL, 0.012 kBq/µL, 81.4 GBq/µmol) and the respective competitor **5** or **6** or endogenous bombesin (BBN) used as internal reference compound (25 µL). The competitor was used in 11 different concentrations, ranging from 0.25 to 500 nM for **5** and **6** or 0.1–250 nM for BBN, whereat a twelfth mixture contained no competitor, to ensure 100% binding of the radioligand. After one hour of incubation at ambient temperature, the suspension was filtered and the cells washed thrice with cold PBS. The filter was punched out and measured by γ-counting. The 50% inhibitory concentration (IC_50_) of **5**, **6** and BBN were calculated by fitting the obtained data via a nonlinear regression analysis using GraphPad Prism Software (v5.04). IC_50_ values were obtained from at least three different experiments, each performed in triplicate.

## 4. Conclusions

The presented synthesis pathway towards MIUs comprising chelator and fluorescent dye, as well as either one or two thiol groups, for click chemistry-based efficient conjugation of appropriately functionalized biomolecules enables the tailored design and synthesis of dually labeled biomolecule monomers, dimers, and tetramers. The obtained MIUs and also their bioconjugates can efficiently be labeled with the positron emitters ^68^Ga and ^64^Cu under very mild conditions using NODA-GA as the chelator; this being advantageous for the modification and labeling of stable but also susceptible biomolecules. Although we were able to confirm that the introduction of negatively charged fluorescent dyes affects the resulting in vitro GRPR affinities of PESIN-based peptide dimers, we could also show that this negative effect can at least partly be compensated for by a higher valency of peptide multimerization.

## Figures and Tables

**Table 1:** Summary of the photophysical properties of the dyes NIR-820 and **1**, the MIUs **2** and **3**, and the bioconjugates **5** and **6**. All measurements were conducted in phosphate buffer solution and the excitation wavelength was λ_ex_ = 650 nm.

Compound	λ_max_ (abs) (nm)	log ε (M^−1^ cm^−1^)	λ_max(em)_ (nm)	Stokes Shift (nm)
NIR-820	785 [30,39]	5.3	805 [30,39]	20
**1**	770 [30,31]	5.4	790 [30,31]	20
**2**	695/770	4.7/4.6	790	20
**3**	695/770	4.9/4.9	795	25
**5**	710/770	4.6/5.0	790	20
**6**	711/775	4.5/5.0	795	20

## Data Availability

The data presented in this study are available on request from the corresponding author.
